# Is the Benefit–Risk Ratio for Patients with Transfusion-Dependent Thalassemia Treated by Unrelated Cord Blood Transplantation Favorable?

**DOI:** 10.3390/ijms18112472

**Published:** 2017-11-20

**Authors:** Tang-Her Jaing

**Affiliations:** Division of Hematology and Oncology, Department of Pediatrics, Chang Gung University and Children’s Hospital, 5 Fu-Shin Street, Kwei-Shan, Taoyuan 33302, Taiwan; jaing001@cgmh.org.tw; Tel.: +886-3328-1200 (ext. 8226); Fax: +886-3328-8957

**Keywords:** transfusion-dependent thalassemia, hematopoietic stem cell transplantation, cord blood transplantation

## Abstract

Transfusion-dependent thalassemia (TDT) is an inherited disorder characterized by absent or defective production of α- or β-hemoglobin chains. If untreated, the disease invariably culminates in death in early infancy due to cardiac failure or overwhelming infection. Although there is clear evidence of good health-related quality of life and return to normal life style, the choice to undergo hematopoietic stem cell transplantation (HSCT) remains a challenge because of the potential risk of transplant-related mortality (TRM) in TDT. Successful hematopoietic stem cell transplantation may cure the hematological manifestations of TDT, but introduces risks of TRM and morbidity. The low incidence of graft-versus-host disease (GVHD) provides the major rationale for pursuing unrelated cord blood transplantation (CBT). Considerable evidence suggests a lower rate of recurrence after CBT than after transplantation from adult donors. As the TRM, overall survival, and thalassemia-free survival for CBT improve, the utility of this stem cell source will expand to indications that have hitherto rarely used unrelated CBT. This paper summarizes the current progress in understanding the advances in unrelated CBT for thalassemia. Although as yet only in a limited number of patients, the results of unrelated CBT for thalassemia are encouraging.

## 1. Introduction

The term thalassemia describes a group of inheritable disorders caused by the absence or reduction in globin chain production. This results in ineffective erythropoiesis, anemia, and poor oxygen delivery. Ineffective erythropoiesis is associated with bone expansion and extramedullary hematopoiesis in the liver, spleen, and other sites. Transfusion for severe anemia and the maintenance of hemoglobin levels of at least 90–100 g/L allows for normal growth and development, and serves to suppress ineffective erythropoiesis, hepatosplenomegaly, and bone deformities. Hemosiderosis occurring as a result of frequent transfusions and enhanced gastrointestinal iron absorption can cause severe iron overload and deposition in organs such as the heart, liver, and endocrine glands, and eventually lead to multiorgan failure. Severe endocrinopathies often require hormone replacement; however, severe cardiac toxicity is life-threatening, and is the main cause of death in thalassemia patients [1].

Iron chelation therapy is largely responsible for doubling the life expectancy of patients with thalassemia major [2]. Currently, life expectancy for these patients can reach 50 years and over. The Pesaro experience has clearly defined three iron-related factors that significantly affect transplant outcomes [3]. Patients can be stratified according to the Pesaro risk factors: hepatomegaly (≤2 cm versus >2 cm from the cost arch), liver fibrosis (absent versus present to any degree), and duration of exposure to iron chelation with quality of chelation treatment given before transplant (Table 1). Despite increased knowledge, there are still uncertainties regarding the level of body iron at which iron chelation therapy should be initiated, as well as the appropriate degree of iron store depletion [4].

Hypertransfusion and iron chelation have been proven to improve life expectancy and quality of life (QOL). However, hypertransfusion can be associated with the risks of alloimmunization and blood-transmitted infections, especially hepatitis B and C, in areas where blood safety is poor. Moreover, iron chelation is prohibitively expensive for patients in most regions where thalassemia is prevalent, and compliance in patients from developed countries is frequently poor. Nevertheless, thalassemias are a global health burden due to population migration and growth as well as improved survival leading to an increase in the incidence of the disorder [5].

## 2. Iron Chelation Therapy and Adherence

In patients who are not receiving transfusions, abnormally regulated iron absorption results in increases in the body’s iron burden, depending on the severity of the erythroid expansion. Regular transfusions may double this rate of iron accumulation. Iron deposition occurs in visceral organs, causing tissue damage and ultimately organ failure. Cardiac events due to iron overload are still the primary cause of death in patients with transfusion-dependent thalassemia (TDT). It has been postulated that labile plasma iron (LPI) could play a role in the increased apoptosis of developing precursors in the bone marrow, leading to ineffective erythropoiesis [6]. LPI is membrane-permeant, and causes subsequent cell damage. Both transfusional iron overload and excess gastrointestinal absorption contribute to iron accumulation. The excess gastrointestinal iron absorption persists despite a massive surge in total body iron load. Regularly transfused patients with TDT are exposed to transfusion-related iron overload, which can lead to iron toxicity, with organs such as the heart, liver, and endocrine glands being particularly vulnerable. Iron overload is the major cause of morbidity and mortality in thalassemia [7]. Improvement of growth after HSCT in TDT is common. Gonadal failure is universal in girls, while boys were less affected [8].

Research suggests that iron chelation therapies impact QOL and result in low levels of personal satisfaction [9]. Iron-chelating agents are used for preventing and treating iron overload. Deferoxamine (DFO) has been the standard treatment for the last 40 years; it is administered subcutaneously or intravenously usually over 8–12 h, for up to 7 days a week. There are currently two oral iron chelators—deferiprone and deferasirox—licensed for the treatment of iron overload. These were initially introduced as second-line agents for children aged ≥6 years with β-thalassemia major or individuals in whom DFO is contraindicated or found to be inadequate [10]. These oral agents are becoming more commonly used, particularly deferasirox, because of the ease of administration compared with subcutaneous or intravenous DFO [4]. The price of therapy varies depending on the formulation and the dose prescribed, but treatments can cost in excess of £1000 per month. However, the intensive demands and uncomfortable side effects of iron chelation therapy can have a negative impact on daily activities and well-being, which may affect adherence to therapy.

With the improvements in transfusion management, infectious disease screening, and innovations in oral iron chelation, hematopoietic stem cell transplantation (HSCT) is still the only curative therapy for TDT. For many patients, cure of their disease is the ultimate goal, sometimes because of the failure of their medical support therapy or simply because of a desire for a normal life without chelation or transfusion. Since the first successful curative transplant in a child with thalassemia major by Thomas and colleagues at the Fred Hutchinson [11], and the decades of optimization by the Italian groups [12], more than 3000 thalassemia patients have received transplantations worldwide, mostly using human leukocyte antigen (HLA)-identical sibling donor bone marrow. The Pesaro experience has demonstrated that the overall survival and thalassemia-free survival after HSCT from an HLA-identical donor ranges from 87% to 95% and from 64% to 90%, respectively, depending on the Pesaro risk class [13]. Cord blood transplantation (CBT) has the theoretical advantages of rapid availability and low risk of severe graft-versus-host disease (GVHD), despite donor–recipient HLA disparity.

## 3. Biological Characteristics of Unrelated Cord Blood and Implications for Transplantation

However, fewer than 30% of patients have unaffected HLA-matched siblings. Matched unrelated adult donors remain unavailable for most TDT patients, despite proving to be an acceptable alternative for patients with thalassemia who lack a compatible family donor. Unrelated umbilical cord blood may alleviate the shortage of matched unrelated donors, considering that less stringent HLA matching is acceptable. Because of the easier accessibility and less severity of GVHD, unrelated cord blood (UCB) has been increasingly used as an alternative to bone marrow for HSCT [14]. CBT may be preferable to transplantation from adult donors for thalassemia and other non-malignant diseases, because the potential long-term elimination of GVHD prophylaxis greatly improves the QOL for transplant patients compared with medical therapy alone.

As in the context of malignant diseases, GVHD offers no advantage of relapse reduction for thalassemia and other transplant patients with non-malignant diseases. In a retrospective single-center study of 724 thalassemia patients, Gaziev et al. observed no difference in acute GVHD incidence among patients in the three Pesaro classes, but there was a remarkable and statistically significant difference in mortality from grade III and IV GVHD (27%, 48%, and 84% in the three Pesaro classes; *p* < 0.0001) [15].

UCB contains lymphoid and dendritic cells as well as cells of hematopoietic lineages. In addition, UCB units contain variable percentages of cells of maternal origin, a phenomenon called maternal microchimerism [16]. From an immunological standpoint, pregnancy represents an extraordinary situation in which both the fetus and mother are exposed to an immunologically foreign organism. CD4+ CD25+ FoxP3+ T regulatory (Treg) cells dominate the fetal immune system during mid-gestation, with numbers declining toward adult levels by the time of delivery [17]. It is likely that the powerful suppressive effect of fetal Treg cells contributes at least partially to the suppression of GVHD reactions after UCBT.

Umbilical cord dendritic cells are hyporeactive upon stimulation, with the limited upregulation of surface receptors, limited signaling, and a bias against inducing CD4+ T helper 1 responses [18]. The naiveté of UCB lymphocytes, however, results in delayed immune reconstitution and infection-related mortality in transplant recipients. The defective capability of UCB dendritic cells to stimulate naive T cells and initiate a primary immune response may contribute to the infection susceptibility during the late post-transplant period [19].

## 4. Hematopoietic Potential of UCB: Comparison with Adult Stem Cells

Although the past few decades have shown an improvement in the survival and complication-free survival rates among patients with β-thalassemia major and gene therapy is another option on which scientists are working, due to vector-associated limitations, they have limited utility in hemoglobinopathies [20]. If gene therapy is to provide a cure, it needs to obtain equivalent results in terms of cost/benefit ratio with HSCT. HSCT continues to be the only effective and realistic approach to the cure of this chronic non-malignant disease. A study in a large cohort of ex-thalassemia patients who underwent HSCT more than 20 years previously revealed that the ex-patients, their sibling donors, and the general population had a very similar QOL, and the QOL was better in the ex-patients than in a control group of thalassemia patients treated conventionally with blood transfusions and iron chelation therapy [21]. Currently, the availability of an international network of voluntary stem cell donor registries and cord blood banks has significantly increased the odds of finding a suitable HLA-matched donor [22]. No prospective randomized clinical trial will be able to provide a definitive answer to the challenge of choosing between CBT and medical therapy for each individual patient. For pediatric patients, parents face an even more difficult decision. Transplanted TDT patients enjoy a better QOL, mainly in physical health, than do conventionally treated patients. For patients, families, and referring and transplant physicians to accept unrelated CBT for TDT, the benefit–risk ratio has to be significantly improved so that it is worthwhile for patients to take a chance on a risky procedure to prolong the lifespan or improve the QOL.

Various other approaches have been tried, and some have been proven to improve the outcome of CBT for thalassemia with related HLA-identical donors [23,24]. Patients with TDT have excellent outcomes after both HLA-identical sibling CBT and bone marrow transplantation. Unrelated CBT is not widely used to treat hemoglobinopathies despite being the fastest growing stem cell source for unrelated HSCT. Published series have shown unfavorable disease-free survival [25], or were single-institution efforts [26]. Moreover, within the first 100 days, the absolute costs of CBT are usually higher than matched related donor transplantation. These costs are primarily driven by severe post-transplant complications, graft failure, and prolonged inpatient stay [27]. Strategies to enhance the engraftment of unrelated donor marrow or UCB-derived hematopoietic stem cells (HSCs) will decrease the costs of HSCT. So far, more than 300 thalassemia patients have been transplanted from an unrelated donor. Table 2 illustrates the outcome of unrelated HSCT, including CBT, performed to cure children and young adults with β-thalassemia major, using both myeloablative or reduced-intensity conditioning regimens.

The phenotypic characteristics of UCB and adult bone marrow cells are remarkably similar [28], with the exception of a higher density of expression of CD34 on the cells and an increased expression of HLA-DR on neonatal cells. Functionally, UCB has a higher proportion of cells with stem cell characteristics such as the ability to re-plate and expand while maintaining primitive characteristics. The mechanisms underlying the proliferative and expansive advantages of UCB over adult HSC include a longer telomere length, a more rapid exit from G_0_/G_1_ into the cell cycle, increased cytokine sensitivity, and/or paracrine cytokine effects. In clinical practice, the usual measure of HSCs is the number of CD34+ cells in the graft. UCB grafts contain at least one log fewer CD34+ cells than do adult CD34+ stem cell grafts. The low number of HSCs in UCB grafts explains the often slow and erratic hematopoietic recovery.

## 5. Conclusions

Not all centers have experience in all types of transplants from different sources or with diverse conditioning intensities. Current research is focused on the use of UCB as an alternative stem cell source in unrelated transplantation for thalassemia. In a pediatric setting, this option has the advantage of being immediately available when required, and UCB is not as difficult to match as are stem cells from an adult volunteer donor. The incidence of GVHD is widely variable for acute and chronic forms. Unfortunately, the rejection rates remain high. Although in a limited number of patients, the results of unrelated CBT for thalassemia are encouraging. An in-depth comprehension of the immunogenetic factors associated with a reduced risk of GVHD and TRM may possibly assist us in the selection of ideal donors for thalassemia patients. In the context of a modern risk- and subset-oriented therapy, early transplantation is of utmost importance and therefore needs to be performed by well-trained and highly experienced personnel.

## Figures and Tables

**Table 1:** Pesaro risk factors and risk classes for allogeneic hematopoietic stem cell transplantation in thalassemia.

Risk Classes	Hepatomegaly	Liver Fibrosis	Chelation History
Class 1	No	No	Regular
Class 2	No/Yes	No/Yes	Regular/Irregular
Class 3	Yes	Yes	Irregular

**Table 2:** Clinical outcomes of unrelated hematopoietic stem cell transplantation in young adult and pediatric thalassemia patients.

First Author	No Patients	Source	Age Median Years (Range)	OS (%)	TFS (%)	TRM (%)	Rejection (%)	aGVHD (%)	cGVHD (%)	Reference
La Nasa	32	BM	14 (2–28)	79	66	19	12.5	41	25	[29]
La Nasa	68	BM	15 (2–37)	79.3	65.8	20	13	40	18	[30]
Hongeng	21	BM	4 (0.7–12)	85.7	71	14.3	14.3	42	14	[31]
Locatelli	122	BM	10.5 (1–35)	84	75	16.4	13.1	28	13	[32]
Ruggeri	35	CB	4 (0.5–14)	62	21	34	57	23	16	[24]
Jaing	35	CB	5.5 (1.2–14)	88.5	88.5	11.4	14.4	47	35	[25]
Li	52	BM/PB	6 (2–15)	92.3	90.4	7.7	1.9	9.6	0	[33]
Anurathapan	26	BM/PB	8 (2–10)	94	82	7	0	28	15	[34]
Shah	9	CB	3.8 (1.5–7)	100	56	0	44	33	11	[35]

OS: overall survival; TRM: transplant-related mortality; TFS: thalassemia-free survival; aGVHD: acute graft-versus-host disease; cGVHD: chronic graft-versus-host disease; BM: bone marrow; CB: cord blood; PB: peripheral blood.

## Abbreviations

CBT	Cord blood transplantation
GVHD	Graft–versus–host disease
HSCT	Hematopoietic stem cell transplantation
LPI	Labile plasma iron
QOL	Quality of life
TDT	Transfusion-dependent thalassemia
TRM	Transplant-related mortality
UCB	Unrelated cord blood
